# A dataset of scientific citations in U.S. patent Office Actions

**DOI:** 10.1038/s41597-026-06720-7

**Published:** 2026-01-31

**Authors:** Kyle Higham, Hannah Kotula, Emma Scharfmann, Steve Gong, Gaétan de Rassenfosse

**Affiliations:** 1 Motu Economic and Public Policy Research, Wellington, 6011 New Zealand; 2 University of California, Berkeley, College of Engineering, Berkeley, 94720 USA; 3 Google, Mountain View, 94043 Mountain View, USA; 4 College of Management of Technology, École polytechnique fédérale de Lausanne, Lausanne, 1015 Switzerland

**Keywords:** Intellectual-property rights, Intellectual-property rights

## Abstract

We present a curated dataset of about 850,000 citations extracted from Office Actions issued by examiners at the United States Patent and Trademark Office. These references, historically underused due to accessibility challenges, provide a granular view into the patent examination process and complement traditional front-page citation data. We classify each citation into one of 14 categories and focus on the 265,000 references to scientific literature, which we parse, clean, and disambiguate using machine learning and external bibliographic services. To enhance reusability, disambiguated records are linked to OpenAlex, a comprehensive research metadata platform. The dataset enables new research on examiner behavior, science-technology linkages, and the construction of citation-based metrics. All data and code are openly available to facilitate reuse across disciplines.

## Background & Summary

Patents are a rich source of publicly accessible technical knowledge, offering a unique perspective on the development and dissemination of technological innovations across time and regions. Patent data have been widely used in both academic research and policy analysis to explore a diverse array of topics spanning multiple disciplines. Reflecting their broad utility, numerous initiatives have aimed to clean, disambiguate, and interlink patent datasets to enhance their usability *e.g*.,^1–4^.

Among the various features of patent data, citations play a particularly critical role. They document relationships between inventions, providing a record of how innovations build upon or are influenced by prior developments^5,6^. Researchers have leveraged patent citations to create indicators of patent importance, ranging from basic citation counts to advanced metrics such as originality and network-based measures. These citations have also been instrumental in analyzing the diffusion of innovation^7–9^. A Google Scholar query of the term ‘patent citations’ returns approximately 18,000 research papers since the year 2000.

Despite their apparent simplicity, citation data are the product of a complex process that is often overlooked in research. Traditionally, studies rely on front-page citations, which appear on the first page of granted patent documents. To understand the origin of front-page citations, it is helpful to outline the journey of a patent application at the U.S. Patent and Trademark Office (USPTO), from drafting and filing to prosecution and issuance.

Patent drafting is typically carried out by patent attorneys who, after consulting with inventors, may include citations to patent and non-patent references. These citations often provide context for the invention or acknowledge prior art, with input from inventors being common but not guaranteed. Such references, embedded in the patent document’s text, are known as in-text citations^10,11^.

At the time of filing the patent application to the patent office, the patent attorney must file an ‘Information Disclosure Statement’ (IDS). It is a comprehensive list of potentially relevant prior art that the applicant is aware of— a ‘suggestion list’ that may help the examiner assess the patentability of the invention. Notably, not all references in the IDS appear in the patent’s text and vice-versa.

During prosecution, the patent examiner evaluates the application against criteria such as novelty and non-obviousness. This task involves prior art searches and official communications, known as Office Actions (OAs), where the examiner explains the acceptance or rejection of claims, often citing relevant prior art. Applicants may respond by amending claims, arguing against rejections, or both, in a process that continues until the patent is granted or abandoned. OA citations thus represent snapshots of the examiner’s reasoning at specific points in time during the prosecution process. Once a patent is issued, the front-page citation list is finalized, comprising references deemed most relevant after examination. These references are flagged as either applicant- or examiner-added.

Four important implications regarding front-page citations follow. First, front-page citations identified as arising from the ‘examiner’ may actually correspond to applicant-submitted references retained by the examiner. Using front-page data alone, there is no way of knowing whether the examiner independently found the citation or whether the applicant initially suggested it. Second, not all citations submitted by the applicant through the IDS make it to the front page. In that sense, the set of ‘applicant’ citations does not necessarily represent the full set of citations submitted by the applicant. Third, abandoned (or ‘refused’) patent applications lack front-page citations altogether. Fourth, even for granted patents, front-page citations exclude prior art that led to the rejection of claims during prosecution.

OA citations address some of these shortcomings: they are available for abandoned patent applications, cover all claims in the original patent application, and contain all references that the examiner officially communicated to the applicant. For these reasons, they offer unique insights into the prosecution process and the examiner’s evaluation of prior art. Yet, researchers have largely ignored them due to their lack of accessibility.

In this paper, we offer a pipeline to parse and disambiguate OA citations and share the resulting dataset as a new resource for the analysis of patent data. While the USPTO already provides structured patent-to-patent OA citations through its enriched citation pipeline, no such infrastructure exists for non-patent references. We, therefore, focus on the more challenging task of identifying and disambiguating citations to scientific literature—a growing area of interest in recent research^12,13^. Our dataset includes about 850,000 OA citations, systematically classified into 14 distinct reference types, such as journal articles, books, and product documentation. Of these, over 265,000 scientific citations were parsed and disambiguated using machine learning and external bibliographic services. To enhance interoperability, the disambiguated references are linked to OpenAlex, a comprehensive metadata repository for scholarly works.

## Methods

The data generation process consists of five main stages: data acquisition, classification of OA citations, parsing of bibliographic citations, consolidation, and final data assembly. We describe the five stages below.

### Data Acquisition

We used publicly available patent data from the USPTO, hosted on Google Cloud. Specifically, the source data originates from the USPTO ‘Enriched Citation API’ available at https://developer.uspto.gov/api-catalog/uspto-enriched-citation-api-v2. These data are stored in the Google BigQuery platform under the following fully qualified table name: patents-public-data.uspto_office_actions_citations.enriched_citations. The table contains Office Actions mailed since October 2017. Because some patents take a long time to be issued, their filing dates can predate active prosecution by several years. In our sample, the earliest patent was filed in 2008 and the latest in 2023.

We join this table to the cross-walk dataset, which establishes a correspondence between the cited_Document_Identifier and patent publication numbers in the standard DOCDB format. The crosswalk table name is citedoc_pub_crosswalk. The cited_Document_Identifier serves as a unique identifier for citations found in an examiner’s OA communication.

The crosswalk dataset comprises 6,136,550 entries, of which 5,286,677 are associated with a DOCDB publication number, indicating that these citations correspond to patents. The remaining 849,873 entries lack a matched patent publication number. These unmatched records typically represent citations to prior art outside the patent literature, officially categorized as non-patent literature (NPL) references. These NPL references formed the starting set for our consolidation efforts.

We performed two minor pre-processing tasks before classification. First, we addressed some rare inconsistencies in the NPL references, as some entries pointed to patent literature but were not successfully consolidated in the original dataset. Specifically, we identified and categorized entries beginning with terms such as ‘database wpi’ (8 entries), ‘patent abstract’ (3 entries), ‘chemical abstract’ (274 entries), and ‘wpi’ (3 entries). These entries point to patent databases rather than NPL.

For example, one such reference is: DATABASE WPI, Week 201155, Thomson Scientific, London, GB; AN 2011-D34107, XP002778871, & KR 100 023 792 B1 (PARA CO LTD) 21 March 2011 (2011-03-21) * abstract *. This citation refers to Thomson Scientific’s World Patent Index (WPI), a database used for patent searches and prior art references. The code ‘AN 2011-D34107’ is the WPI accession number (a unique identifier for the entry), and the citation relates to a South Korean patent document filed by PARA CO LTD. The abstract of this patent served as the relevant portion considered for prior art evaluation.

Second, we cleaned the raw citation strings by converting text to title case, trimming whitespaces from the start and end of the strings, and removing control characters, such as non-printing characters like SOH (Start of Heading). After removing duplicates, we obtain 846,800 ‘unique’ citation strings—some of them possibly pointing to the same references.

### Classification

Patent examiners consult a wide range of sources to identify relevant prior art. While patent documents constitute the vast majority of references, examiners also rely on scientific publications and other material they consider pertinent, such as websites or product documentation.

To better understand the nature of these references, we systematically reviewed the set of OA citations in our dataset and developed a classification scheme based on observed patterns. We identified 14 distinct categories, listed here in alphabetical order: Books, Conference proceedings, Databases, Journal articles, Litigation cases, Office actions, Patent documents, Preprints, Product documentations, Search reports, Standard documents, Theses, Webpages, and Wikipedia articles.

To automate the classification of OA references, we developed a machine learning classifier that assigns each reference to one of these classes. We began by labeling 5,000 references using GPT-4 and assessing GPT-4’s precision using 300 random references that we manually classified. After some prompt engineering, GPT-4 classified these citations very accurately but also involved significant costs to classify hundreds of thousands of sentences. Therefore, we have used GPT-4 labels as a training set to fine-tune our own classification model. Specifically, we fine-tuned a multilingual BERT model^14^ using these 5,000 labeled references as the ground truth. The fine-tuned model was then used to classify the 846,800 extracted citations into the 14 classes we enumerated above.

For the remainder of the analysis, we focus on scientific references, which we define as those falling into the following five classes: Books, Conference proceedings, Journal articles, Preprints (including working papers and technical reports), and Theses. There are 265,880 such references.

### Parse Citations

Having identified the scientific citations among the set of NPL references, we then parsed the raw citation string to facilitate the consolidation task. We have relied on Grobid, a machine-learning library for extracting, parsing, and re-structuring raw inputs^15^. Grobid assigns elements in the raw citation string to predefined fields, including, but not limited to, title, publication year, and journal. We interacted with Grobid (v0.7.3) via the Python Grobid client and Docker. (Note that Grobid could not process 0.765 percent of the citations in our sample for reasons that are not immediately apparent upon inspection of problematic citations).

Table 1 provides an overview of the piece of information recovered for the 263,767 scientific references submitted to Grobid. For instance, we recovered the title for 72.26 percent of the references and the ‘complete’ metadata for 30.04 percent of the references. However, we do not necessarily need the complete metadata to consolidate the reference. To illustrate the point, consider, for instance, *Buschmann Et Al., “Levenshtein Error-Correcting Barcodes For Multiplexed Dna Sequencing”, Bmc Bioinformatics*, and *Ridker (2005) Nejm 352 :20-8*. Grobid failed to identify the publication year in the first example and the paper title in the second example (understandably so). Yet, we were able to find to full references based on these partial metadata.

**Table 1:** Number of scientific references, by piece of information.

	Count	Percent
title	190,610	72.26
author_id	203,569	77.18
journal_id	97,354	36.91
year	238,716	90.5
volume	170,689	64.71
issue	54,799	20.78
first_page	166,744	63.22
last_page	166,744	63.22
meta_data	79,234	30.04
title_and_or_meta_data	215,338	81.64

Note 1: N = 263,767.

Note 2: ‘meta_data’ refers to a citation having first author_id, journal_id, publication year, and at least one of: volume number, issue number, first_page number, or last_page number.

Before moving to the consolidation task, we carried out the following pre-processing steps:

- Title. We removed all non-alphanumeric characters.
- Author. We removed ‘*et al*’.
- Year. If the extracted year string was longer than four characters, we extracted the first four characters. We do so because Grobid sometimes incorrectly extracts the year (*e.g*., 2013-07-05 rather than 2013).
- Page numbers (first page and last page). We removed all non-numeric characters. We made this decision because Grobid sometimes incorrectly extracts the page number (*e.g*., *A*272911 rather than 272911).
- If year, page number, volume, or issue was not numeric, we assigned it to missing.

### Consolidate Citations

Consolidation involves leveraging external bibliographical services to correct and enhance citation data. In this study, we relied on OpenAlex and Crossref for consolidating the scientific references.

OpenAlex is a comprehensive, free catalog of the global research ecosystem, indexing over 243 million works. It offers approximately twice the coverage of other services, such as Scopus and Web of Science, and provides significantly better representation of non-English works. We accessed OpenAlex data programmatically using its REST API, implemented in Python with the requests package. Crossref offers metadata, including DOIs, for 165 million works. While Crossref requires no additional setup, it is limited by a query rate of approximately 25 requests per second, posing challenges when scaling to large datasets.

Given these considerations, we selected OpenAlex as our primary tool for consolidating citations due to its extensive coverage and ease of use. For citations that OpenAlex could not consolidate, we supplemented our approach with Crossref.

**Fig. 1:**
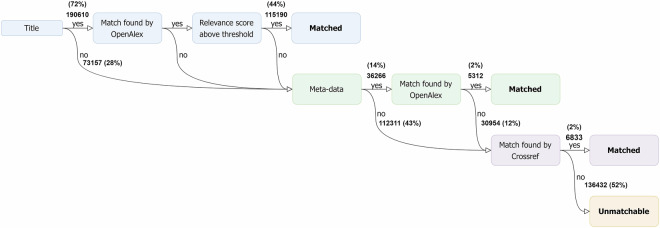
Pipeline of Consolidation Process.

Figure 1 provides an overview of the consolidation process. If a citation contained a title, we searched in OpenAlex using the title. If a citation did not contain a title (or a match was not found using the title), we searched using the metadata. We also used the metadata when the ‘relevance score’ of a title-matched citation, a measure provided by OpenAlex, was below 600. We selected 600 as our threshold based on validating a sample of 100 title-matched citations.

The search using metadata works as follows. We started by filtering out citations that were missing information for the first author, journal, or publication year. To ensure a low false positive match rate, we further limited our sample to citations that contained at least one of the following pieces of information: volume number, issue number, first page number, or last page number. OpenAlex has a unique identifier for each author and journal. We searched for these unique identifiers, and only proceeded if a unique identifier was found for both the first author and the journal. Next, we used these unique identifiers along with publication year and any extra information (namely, volume number, issue number, first page number, last page number) to search OpenAlex. Because volume number, issue number, and page numbers are not formatted consistently across citations, Grobid may have incorrectly assigned these values in the parsing stage (*e.g*., volume and issue number could be swapped around). For this reason, we searched OpenAlex using all permutations of the values assigned to these four pieces of information. Note that we permuted the values for all fields (even if a value for a given field was missing). Unlike title searches, these searches do not return a relevance score. For this reason, we selected the first result returned by OpenAlex, as we are unable to determine the best match in cases where more than one permutation returns a match—however, we note that this outcome is very rare given the strict filtering criteria described above.

Finally, if a match was not found using OpenAlex, we used Grobid to extract and consolidate the citation using Crossref. If this last step was successful, *i.e*., a DOI was found, we sent the DOI to OpenAlex to retrieve the meta-data for the citation.

### Final Data Assembly

We then expanded the consolidated citation data by merging them with publicly available patent data, namely the ‘enriched’ dataset from the USPTO (discussed above) and metadata sourced from OpenAlex.

## Data Records

### Data files

The resulting data are available as two tables stored in CSV files, *final*_*data*_*v*2. *csv* (883.75 MB) and *oa*_*citations*_*classified*_*v*2. *csv* (94.62 MB), available from Figshare^16,17^. The file *final*_*data*_*v*2. *csv* contains the expanded data for each OA reference. The file *oa*_*citations*_*classified*_*v*2. *csv* contains the class label of each OA reference.

### Data and variables

The file *final*_*data*_*v*2. *csv* contains 142,614 entries (excluding the first row, containing the column labels), each representing a unique pair ‘scientific OA citation - patent application number.’ Table 2 presents the list of variables. The metadata use the same labels as OpenAlex and are often self-explicit. They include, among others, the publication year, the journal name, and information on authors, such as institutional affiliations details. Because there could be many authors that could themselves have many affiliations, the number of rows is fairly large. The file allows for up to 112 authors with up to 23 institutions per author. Note that the OpenAlex output contains a greater number of variables than the ones provided in the CSV file. The full OpenAlex output can easily be retrieved by using the openalex_id variable.

**Table 2:** List of variables.

Variable name	Description	From
md5_id	MD5 hash	Own
label	Class label	Own
citedDocumentIdentifier	Identifier for the reference cited in the OA	USPTO
patentApplicationNumber	Patent application number	USPTO
officeActionCategory	Office action category, *e.g*., non-final OA or final OA	USPTO
examinerCitedReferenceIndicator	References listed by examiner in a standard USPTO 892 form in the application	USPTO
citationCategoryCode	X, Y, or A as defined in the Manual of Patent Examining Procedure	USPTO
applicantCitedExaminerReferenceIndicator	References listed by the applicant in an IDS in the application	USPTO
groupArtUnitNumber	Art unit that handled the applications	USPTO
openalex_id	OpenAlex ID	OpenAlex
raw_ref	Raw citation string	OpenAlex
publication_year	Publication year	OpenAlex
is_oa	Boolean: True if this work is Open Access	OpenAlex
journal	Journal	OpenAlex
relevance_score	Based on text similarity to the search term. Only defined for title-matched results.	OpenAlex
type	String: The type of the work	OpenAlex
type_crossref	String: Legacy type information, using Crossref’s “type” controlled vocabulary.	OpenAlex
cited_by_count	Integer: The number of citations to this work.	OpenAlex
is_retracted	Boolean: True if we know this work has been retracted.	OpenAlex
auth_position_i	Author position (i)	OpenAlex
auth_id_i	Author ID (i)	OpenAlex
auth_name_i	Author name (i)	OpenAlex
is_corresponding_i	Boolean: If true, this is a corresponding author for this work.	OpenAlex
auth_inst_id_i_j	Author (i) institute ID (j)	OpenAlex
auth_inst_name_i_j	Author (i) institute name (j)	OpenAlex
auth_inst_country_i_j	Author (i) institute country (j)	OpenAlex
auth_inst_type_i_j	Author (i) institute type (j)	OpenAlex

The file *oa*_*citations*_*classified*_*v*2. *csv* contains 846,800 entries (excluding the first row, containing the column labels), each representing a bibliographic OA reference. Table 3 presents the list of variables.

**Table 3:** List of variables.

Variable name	Description
oa_citation	Raw citation string
label	Class label
md5_id	MD5 hash

### Overlap

We have investigated the overlap in scientific citations between front-page and OA citation data for a sample of 50 patents selected at random. A total of four patents (8%) had a perfect overlap, meaning that all the scientific references listed on the front page appeared in the OAs and vice-versa. For 16 patents (32%), the front-page citations missed at least one OA citation. Finally, for the remaining 30 patents (60%), the front-page data fully includes the OA data and also contains additional references.

Note that this result is partly explained by the fact that the number of front-page scientific references is much larger than the number of OA scientific references, averaging 16.38 and 2.26, respectively (with median values of 5 and 1, respectively). Given that the OA citations are rarer than front-page citations and originate directly from the examination process, they are presumably a more powerful signal of prior art relevance.

## Technical Validation

We conducted a series of validation exercises to assess the accuracy of both the classification of OA citations and the consolidation of scientific references. Overall, our classifier achieved a weighted accuracy of 92% across 14 reference categories, with near-perfect accuracy for the most frequent classes such as journal articles and patents. The citation matching process also performed well: using OpenAlex title searches, the correct match rate rose from 70% to 96% across relevance score quartiles, while metadata-based searches achieved a 99% match accuracy. Additionally, we validated the accuracy of citations consolidated via Crossref, confirming the match in 99 out of 100 sampled cases. This section presents the validation exercises that we conducted.

### Validation of classification

**Fig. 2:**
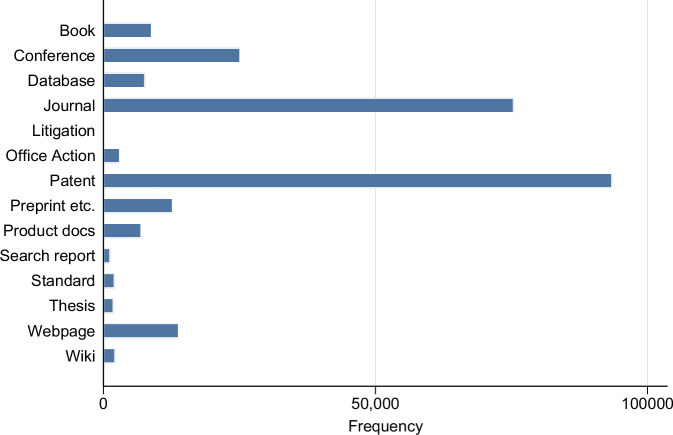
Cited prior art by class, all patents.

As explained, OA citations point to a variety of documents. We have identified 14 classes of documents in the data, but we are interested in those associated with scientific references, namely journal articles, conference proceedings, preprints (including working papers and technical reports), books, and theses. Figure 2 provides the distribution of cited prior art by class. Given that we have excluded OA citations containing a DOCDB number (corresponding to patent documents) from our analysis, it may seem surprising that references to patents form the largest class. Entries in this class correspond to patent documents that the USPTO was not able to disambiguate and, therefore, were not matched to a DOCDB number.

To validate the labeling task, we randomly sampled 420 OA citations and manually checked the accuracy of the assigned class. The sampling is a stratified random sampling of 30 data points per class. Table 4 depicts the results of the validation.

**Table 4:** Validation of classifications.

Class	Accuracy
Book	77%
Conference	43%
Database	23%
Journal	100%
Litigation	33%
Office Action	23%
Patent	100%
Preprint, technical paper, etc.	20%
Product documentation	83%
Search report	73%
Standard	73%
Thesis	73%
Webpage	47%
Wikipedia	67%
Overall	92%

We define accuracy as the fraction of observations assigned to class *x* that are, indeed, true members of class *x*. The overall accuracy score is the average of accuracy estimates for each class, weighted by the proportion of observations in each class. Despite low accuracy in certain classes, the overall score reaches 92 percent because of the predominance of observations in the classes ‘Patent’ and ‘Journal,’ where accuracy reaches 100 percent.

Table 4 nevertheless indicates that some rarer categories remain challenging for the current model. Several low-frequency classes are identified with limited precision in our manual audit (*e.g*., conference papers, databases, and preprints/technical papers). Users should, therefore, treat these fine-grained labels as relatively noisy when analyses hinge on small categories. Where accurate identification matters, we recommend re-training the classifier for these specific classes; the dataset includes the raw citation text to support validation checks and alternative classification approaches. Because the full workflow is open, these rare categories are also straightforward targets for improvement by expanding the labeled training set and re-training with more balanced class representation.

### Validation of OpenAlex ‘title searches’

As mentioned above, when using the title to search OpenAlex, we only consider a citation to be ‘matched’ if the relevance score is greater or equal to 600. To validate the ‘title’ matches, we randomly selected 50 ‘title matched’ citations from each quartile and manually checked them using a Google search. Quartiles are based on the relevance score and were defined using only citations with a relevance score greater than 600. The results are shown in Table 5.

**Table 5:** Validation of OpenAlex ‘title’ matches by quartile. Values are proportions.

Quartile	Correct	No link	Broken link	Incorrect	N
1	0.70	0.20	0.04	0.06	50
2	0.86	0.04	0.02	0.08	50
3	0.86	0.04	0.00	0.10	50
4	0.96	0.04	0.00	0.00	50

The proportion of correctly identified references increases from 70 percent in the lowest quartile to 96 percent in the highest quartile. Failures to identify a correct reference arise for three reasons. First, OpenAlex sometimes does not return a permalink, returning an empty string instead (‘No link’). Second, a permalink exists, but the URL resolves to a non-existent resource (‘Broken link’). Third, the URL resolves to a resource that does not correspond to the actual reference (‘Incorrect’).

### Validation of OpenAlex ‘meta-data searches’

We performed a similar validation exercise for references found through the metadata searches, manually checking 100 of them. Of these 100 citations, 99 were matched correctly. Specifically, the permalink pointed to an existing work, and all the information contained in the citation string (author, year, journal, etc.) matched the work found by OpenAlex.

### Validation of Crossref

Citations unmatched by OpenAlex were sent to Crossref. If Crossref found a DOI, we sent this DOI to OpenAlex to retrieve the meta-data for the citation. Accordingly, three outcomes are possible:

1. Crossref was able to consolidate the citation (*i.e*., a DOI was found) and OpenAlex found a match for this DOI.
2. Crossref was able to consolidate the citation, but OpenAlex was unable to find a match. This could be because the citation was misclassified as scientific.
3. Crossref was unable to consolidate the citation (*i.e*., a DOI was not found).

Table 6 presents the number of observations for each possible outcome. The Crossref approach allows us to recover 6,833 scientific references (Outcome 1). Given that we search OpenAlex with a DOI, if a match is found, it will be correct. However, the DOI found by Crossref may be incorrect (*i.e*., the DOI exists but does not correspond to the raw citation string). For this reason, we validated 100 citations from Outcome 1. We found that all citations were correctly identified (except for one that returned a broken link).

**Table 6:** Overview of the Crossref approach.

Outcome	Count	Proportion
1. Crossref found a DOI and OpenAlex found a match	6,833	0.05
2. Crossref found a DOI, but OpenAlex did not find a match	978	0.01
3. Crossref did not find a DOI	135,454	0.94
Total	143,265	1

Regarding Outcomes 2 and 3, unmatched citations could arise because the citations have been misclassified as scientific references or because the raw string contains insufficient information for a match to be made. We randomly selected 200 unmatched citations (100 from Outcome 2 and 100 from Outcome 3) and counted how many of them were real scientific citations. Of these 200 citations, 23 did not point to scientific references (13 from Outcome 2 and 10 from Outcome 3).

## Usage Notes

This dataset opens new avenues for empirical research at the intersection of science, technology, and intellectual property. First, it provides a granular view of examiner-cited scientific references during the patent prosecution process. Researchers interested in examiner behavior can use the data to ask, for example, how reliance on scientific literature varies across art units or technology areas, whether some examiners introduce scientific references earlier in prosecution than others, and how these patterns differ from applicant-provided citations in the same applications.

Second, the dataset enables a reassessment of citation-based metrics by making the selection process into the final citation record observable. Because Office Action citations can include references that do not appear on the granted patent, it becomes possible to quantify which scientific references are raised during prosecution but later “drop out,” and whether this selection differs across applicants, fields, or time. This allows researchers to test how sensitive common citation-based indicators are to relying only on front-page citations versus incorporating prosecution-stage information, and to interpret citation metrics in light of the examination process that generates them.

Third, the dataset supports work on science-technology linkages in the specific context of examination, especially when used in conjunction with data on inventor affiliations, technology classifications, or grant-level scientific funding. For example, researchers can compare the fields, outlets, or recency of science cited during prosecution to what appears on granted patents, or examine whether prosecution-stage citations point to different parts of the scientific record than applicant disclosures. Unlike datasets focused exclusively on granted patents, this one includes abandoned and rejected applications, which can be useful not only when studying how scientific knowledge enters (or fails to enter) the formal patent record, but also to observe the blocking function of publicly accessible science. We hope that researchers will use our data to answer these questions, and many more.

### Limitations and future refinement

#### Less common document categories

As shown in Table 4, some rare reference types are currently labeled with limited accuracy. Analyses that require accurate identification of specific small categories should validate or refine these labels. Because the full pipeline is open (data, code, and model training workflow), the dataset is designed to be extended and improved upon; for example, by further hand-labeling and model re-training to improve classification of rarer categories.

#### Potential sources of inherited bias

The dataset reflects two upstream processes and therefore inherits their biases: (i) the production and disclosure of scientific references during U.S. patent examination, and (ii) the coverage and linkage properties of the OpenAlex metadata used for document matching. On the examination side, prior work suggests that citation practices can vary systematically across examiner groups, technology domains, and time—for example through examiner ‘home-bias’ effects^18^, limitations and strategy in inventor/attorney search and disclosure practices^19–21^, and temporal ‘collective memory’ or recency effects in what is searched for and cited^22,23^. On the data/linkage side, database coverage and record quality may differ by field, publication venue, language, region, and historical period^24^, which can lead to uneven match rates and differential representation of scientific sources across domains and cohorts. However, we note that, while imperfect, OpenAlex appears to be among the least biased sources along many of these dimensions^25–29^. Furthermore, USPTO examiners only consult English literature, limiting concerns about language bias in our consolidation task. However, language concerns will be important for scholars wishing to extend our work to other offices such as the European Patent Office, which operates with three different languages. Finally, accessibility constraints may shape what is practically discoverable and citable: paywalls, licensing, and institutional access can affect which scientific documents are encountered by applicants, examiners, and third parties, and may correlate with resources and geography^30–33^. Users conducting analyses across technological domains, countries or time should assess robustness to these potential biases.

## Code availabilty

All our code is available on GitHub (https://github.com/moturesearch/office-action-citations).

## Data Availability

The final data files are available from Figshare^16,17^.
